# Are we ‘MPOWERing’ the youth of Africa to stop smoking?

**DOI:** 10.7196/AJTCCM.2019.v25i2.021

**Published:** 2019-07-31

**Authors:** Ismail S Kalla

**Affiliations:** Department of Pulmonology and Department of Critical Care Medicine, Charlotte Maxeke Johannesburg Academic Hospital, University of the Witwatersrand, Johannesburg, South Africa

## Editorial

We have just observed the world No-Tobacco day (31 May, for those of you who may have missed it) with little, if any, fanfare, despite the fact that annually, 8 million deaths are directly attributable to smoking, and 1 million to passive smoking. A more concerning figure is that all forms of tobacco usage are responsible for ~57 million annual deaths worldwide, with tobacco killing 1 person every 4 seconds. Tobacco is deadly in any form, and threatens the lung health of everyone exposed to it.^[1]^

The mortality rates attributable to smoking are frequently quoted. However, the associated morbidity is somewhat neglected. Some of the more common respiratory illnesses directly caused or exacerbated by smoking include tuberculosis (TB), asthma, chronic obstructive pulmonary disease (COPD) and lung cancer. In 2017, 1.6 million people lost their lives as a result of of TB, and 10 million fell ill with the disease.^[2]^ More than 20% of the global TB incidence may be attributable to tobacco usage.^[3]^ Tobacco smoking more than doubles the risk of reactivating TB from a latent state to the active disease.^[4,5]^ School-aged children of adult smokers exposed to the harms of passive smoke are at risk of developing asthma. In asthmatics, smoking is a significant risk factor for frequent exacerbations, and is responsible for approximately 1 in 9 asthma-related deaths.^[6]^ The greatest impact that smoking has in terms of respiratory diseases is in the development of COPD. Approximately one in five smokers will develop COPD, and adults exposed to second-hand smoke during childhood are at increased risk.^[7]^ Tobacco smoking is the single most important aetiology of lung cancer, causing in excess of 1.2 million lung cancer deaths every year.^[8]^ The lifetime risk of developing lung cancer in smokers is 22 times greater than in non-smokers, but more significantly, non-smokers exposed to second-hand smoke have a 30% higher risk of developing lung cancer.^[9]^ Despite the impact that smoking has on respiratory illness, in South Africa (SA), cardiovascular-associated mortality accounts for the majority of deaths associated with smoking, and ~500 000 disability-adjusted life years are lost to smoking-associated illnesses annually.^[6]^

In this issue of the AJTCCM, Batini *et al*. ^[10]^ present data that highlight how far we as a continent are falling behind the rest of the world in introducing smoking cessation strategies. The introduction of the World Health Organization (WHO) Framework Convention on Tobacco Control (FCTC) and the MPOWER strategy to aid governments in tobacco control efforts has had a poor uptake in Africa thus far.^[11]^ The MPOWER strategy was designed to assist in reducing tobacco consumption globally, by: Monitoring tobacco use; Protecting people from tobacco use; Offering help to quit; Warning about the dangers of tobacco; Enforcing bans on advertising and sponsorship; and Raising taxes on tobacco.^[11]^ This WHO initiative has aided developed nations in decreasing the current global tobacco smoking prevalence rates from 27% to 20% between 2000 and 2016. However, there is a striking discrepancy seen in low- v. high-income countries, where governmental buy-in to, and resources directed towards, adopting the FCTC-MPOWER strategies is lacking. In SA we are facing an even greater problem, with the influx of unregulated tobacco products (contraband cigarettes), as well as newer tobacco products that contain chemicals similar to those in traditional tobacco products and are likewise harmful to health.^[12]^

Africa, as one of the largest producers of tobacco, is increasingly targeted by multinational tobacco conglomerates as an emerging market, in order to improve sales as tobacco consumption falls in developed nations. Hence, Batini *et al*. ^[10]^ highlight various problems that Africa faces in introducing smoking cessation strategies, and introduces us to the establishment of a fledgling group of interested parties called ACCESSS: African Collaborative Consortium to Evaluate Strategies to Stop Smoking. SA is among the more compliant of African countries in implementing the WHO smoking cessation strategies. We have access to nicotine replacement therapy, a smoking cessation guideline that is fully endorsed by the SA Thoracic Society and a government that enforces bans on tobacco advertising and frequently increases taxes on tobacco. Yet there is currently only one government-funded smoking cessation clinic in SA.^[13]^ Therefore the aim of ACCESSS will be to increase awareness of smoking cessation strategies, as well as encourage and support clinicians who are interested in smoking-related research. This is a notable endeavour and should receive our wholehearted support.
